# Supplementary figures and images for: Identification of CRF66_BF, a New HIV-1 Circulating Recombinant Form of South American Origin
Source: Front Microbiol. 2021 Nov 15;12:774386. doi: 10.3389/fmicb.2021.774386 (PMC8634668; doi:10.3389/fmicb.2021.774386)

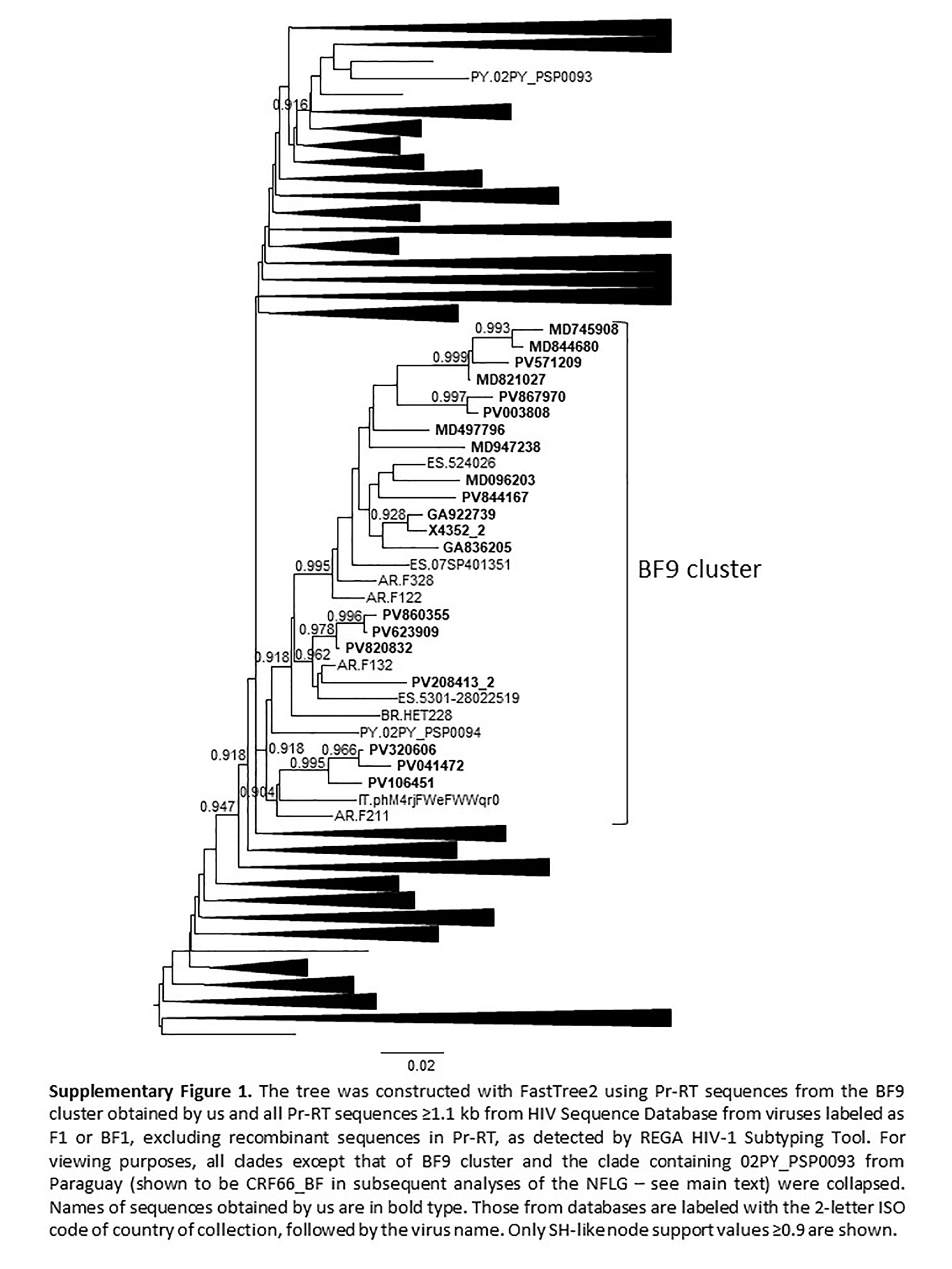

Supplement: Supplementary file 1 [file Image_1.JPEG]

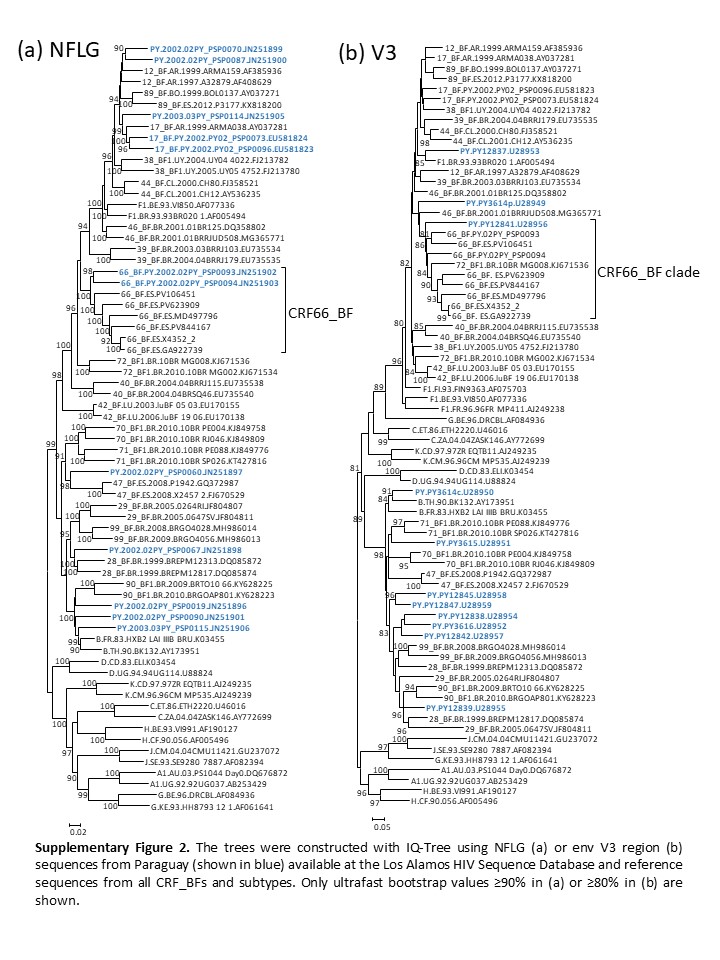

Supplement: Supplementary file 2 [file Image_2.jpg]

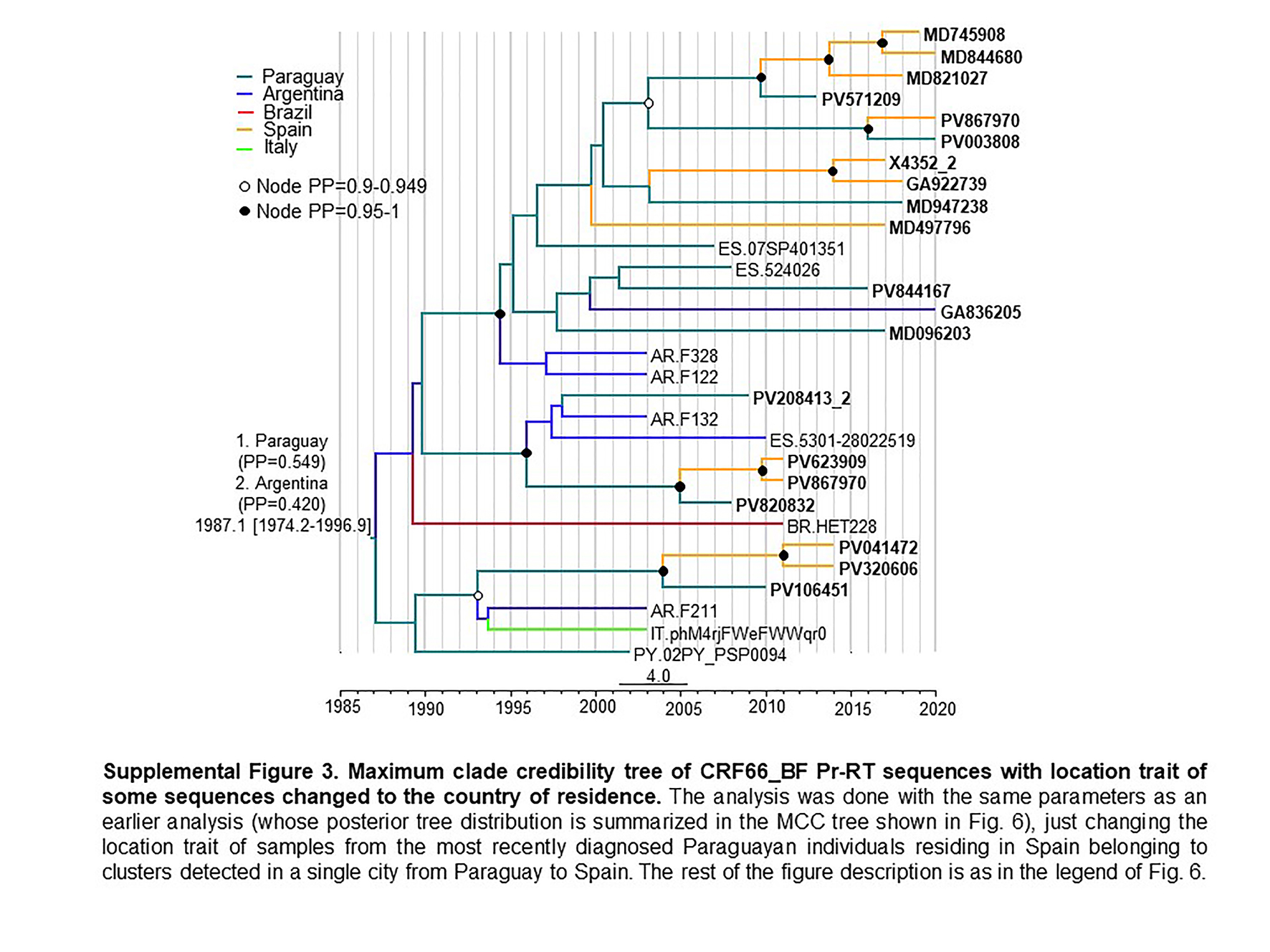

Supplement: Supplementary file 3 [file Image_3.JPEG]

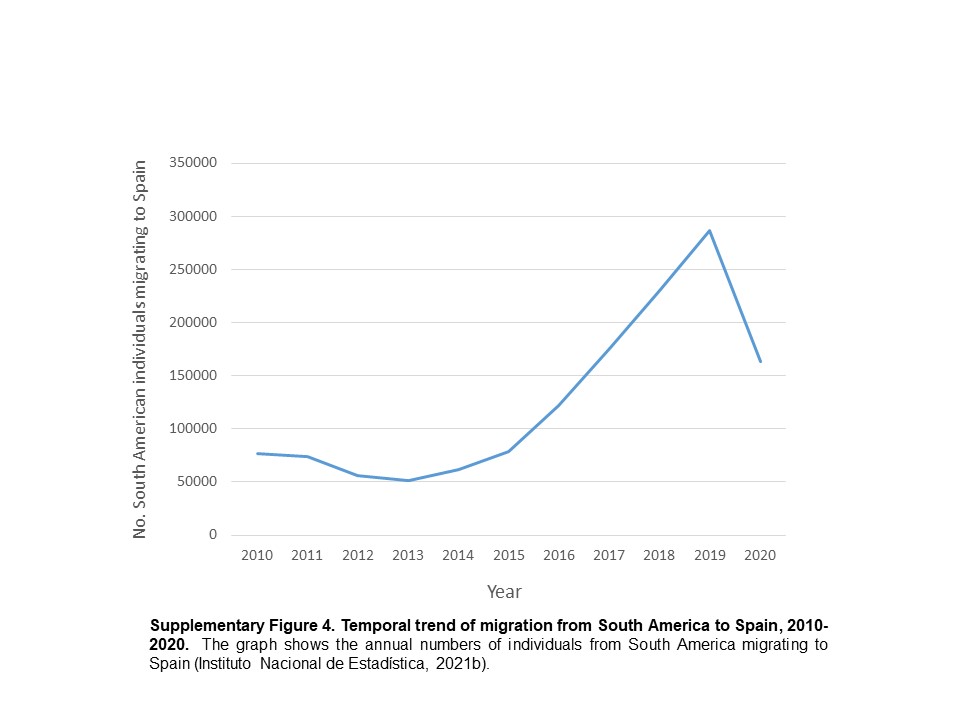

Supplement: Supplementary file 4 [file Image_4.jpg]

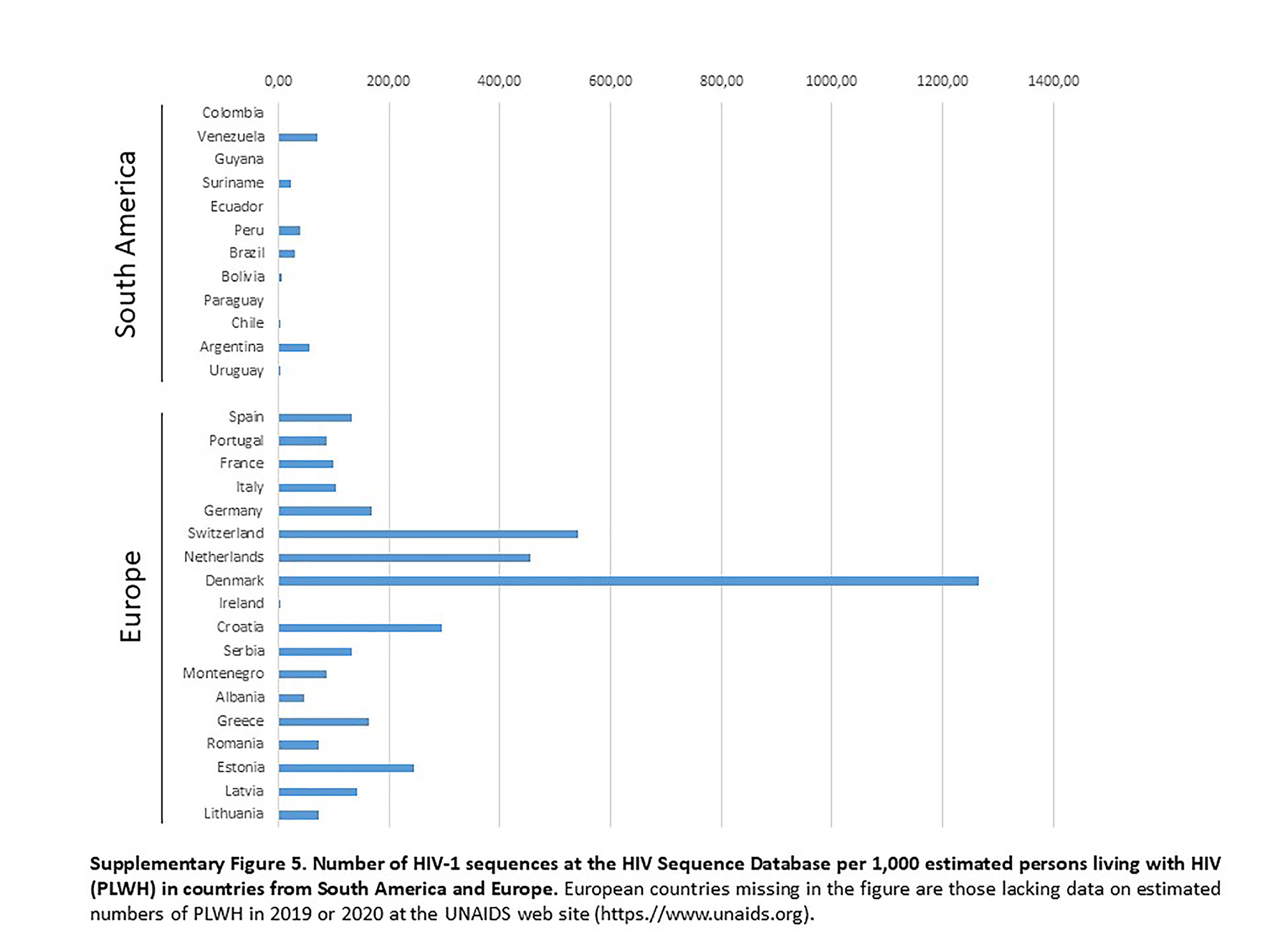

Supplement: Supplementary file 5 [file Image_5.JPEG]
